# Supplementary material for: Altered microRNA Transcriptome in Cultured Human Liver Cells upon Infection with Ebola Virus
Source: Int J Mol Sci. 2021 Apr 6;22(7):3792. doi: 10.3390/ijms22073792 (PMC8038836; doi:10.3390/ijms22073792)
Supplement: Supplementary file 1 [file ijms-22-03792-s001.zip › Supplementary_File/D_ KEGG_Analysis_Results/16-30nt_pathway_Mayinga-96h-Huh7_vs_Control-96h-Huh7_down.mature_mirna_targets/hsa_pathwayResult.html]

| PathwayID | Definition | OriginalWebSite | Fisher-Pvalue | SelectionCounts | SelectionSize | Count | Size | FDR | Enrichment\_Score | GeneRatio | Genes |
| --- | --- | --- | --- | --- | --- | --- | --- | --- | --- | --- | --- |
| hsa00534 | Glycosaminoglycan biosynthesis - heparan sulfate / heparin - Homo sapiens (human) | http://www.genome.jp/kegg-bin/show\_pathway?hsa00534+2135+9394+8509+9348+64132 | 1.556946e-04 | 5 | 176 | 24 | 7906 | 5.200200e-02 | 3.807726 | 0.028409 | EXTL2//HS6ST1//NDST2//NDST3//XYLT2 |
| hsa04918 | Thyroid hormone synthesis - Homo sapiens (human) | http://www.genome.jp/kegg-bin/show\_pathway?hsa04918+23439+821+84699+493869+7849 | 2.420320e-02 | 5 | 176 | 74 | 7906 | 1.000000e+00 | 1.616127 | 0.028409 | ATP1B4//CANX//CREB3L3//GPX8//PAX8 |
| hsa04916 | Melanogenesis - Homo sapiens (human) | http://www.genome.jp/kegg-bin/show\_pathway?hsa04916+84699+1906+2535+7976+2775+3815 | 2.487225e-02 | 6 | 176 | 101 | 7906 | 1.000000e+00 | 1.604285 | 0.034091 | CREB3L3//EDN1//FZD2//FZD3//GNAO1//KIT |
| hsa05020 | Prion diseases - Homo sapiens (human) | http://www.genome.jp/kegg-bin/show\_pathway?hsa05020+712+2002+4684 | 4.217458e-02 | 3 | 176 | 35 | 7906 | 1.000000e+00 | 1.374949 | 0.017045 | C1QA//ELK1//NCAM1 |
| hsa04015 | Rap1 signaling pathway - Homo sapiens (human) | http://www.genome.jp/kegg-bin/show\_pathway?hsa04015+1944+2263+2775+2904+3815+84612+7057+7094+10451 | 4.464826e-02 | 9 | 176 | 210 | 7906 | 1.000000e+00 | 1.350195 | 0.051136 | EFNA3//FGFR2//GNAO1//GRIN2B//KIT//PARD6B//THBS1//TLN1//VAV3 |
